# Convolutional neural network based on photoplethysmography signals for sleep apnea syndrome detection

**DOI:** 10.3389/fnins.2023.1222715

**Published:** 2023-07-21

**Authors:** Xinge Jiang, YongLian Ren, Hua Wu, Yanxiu Li, Feifei Liu

**Affiliations:** ^1^School of Information Science and Electrical Engineering, Shandong Jiaotong University, Jinan, China; ^2^School of Science, Shandong Jianzhu University, Jinan, China

**Keywords:** sleep apnea syndrome (SAS), convolutional neural networks (CNN), photoplethysmographic (PPG) signals, sleep apnea syndrome (SAS) recognition, cross entropy

## Abstract

**Introduction:**

The current method of monitoring sleep disorders is complex, time-consuming, and uncomfortable, although it can provide scientifc guidance to ensure worldwide sleep quality. This study aims to seek a comfortable and convenient method for identifying sleep apnea syndrome.

**Methods:**

In this work, a one-dimensional convolutional neural network model was established. To classify this condition, the model was trained with the photoplethysmographic (PPG) signals of 20 healthy people and 39 sleep apnea syndrome (SAS) patients, and the influence of noise on the model was tested by anti-interference experiments.

**Results and Discussion:**

The results showed that the accuracy of the model for SAS classifcation exceeds 90%, and it has some antiinterference ability. This paper provides a SAS detection method based on PPG signals, which is helpful for portable wearable detection.

## 1. Introduction

According to the statistics of the World Health Organization, more than one-third of the world’s population suffers from sleep disorders, which seriously affect people’s health. SAS is a common sleep disorder, and its standard recognized method of diagnosis is polysomnography. However, this method requires multiple sensors, resulting in discomfort during the detection process. It can also seriously affect the patient’s natural sleep mode, with high costs (Phan and Mikkelsen, 2022). Thus, it is an urgent problem to find a simple and comfortable diagnostic method for the detection of SAS. To improve the comfort of the diagnostic process, thermal infrared imaging, radio frequency (RF) architecture, and sound detection have been introduced for non-contact detection (Murthy et al., 2009; Norman et al., 2014; Penzel, 2017; Tran et al., 2019), since body position, limb movement, and noise can easily interfere with the monitoring results. In recent years, some scholars have been committed to researching SAS detection based on wearable devices, which are used to collect chest bioimpedance, electrocardiogram (ECG), or PPG. At the same time, machine learning or deep learning are used to detect SAS, with accuracy generally around 70–85% (Baty et al., 2020; Hsu et al., 2020; Papini et al., 2020; van Steenkiste et al., 2020).

At present, many scholars have conducted research on convenient SAS detection based on neural networks. Convolutional neural networks have been gradually applied to analyze sleep quality. Song and other researchers constructed convolutional neural networks to classify sleep stages using single-channel electrocardiogram signals (Song et al., 2016; Sors et al., 2018; Wang et al., 2019; Eldele et al., 2021; Haghayegh et al., 2023). Guo et al. (2022) proposed a pseudo-3D convolutional neural network method to detect people’s nocturnal sleep behavior, with an accuracy of 90.67% on the test set. du-Yan et al. (2022) used convolutional neural networks to analyze sleep stages using heart rate variability. Casal et al. (2022) constructed a time convolutional network and transformer using pulse oximeter signals to classify sleep stages. The above research methods mostly extract classification features from ECG signals. However, ECG signals are easily affected by low-frequency and large-amplitude P and T waves, and the above studies are mostly used for sleep stages but not for SAS detection.

Pulse signals contain all kinds of human information and can be easily obtained, and monitoring it is of great significance in assessing the risk of various diseases (Allen and Hedley, 2019). Pulse wave amplitude and pulse rate variability have been used for SAS diagnosis and detection (Haba-Rubio et al., 2005; Liu, 2017). However, when the signal is disturbed or weak, it is very difficult to extract local features of the waveform using these methods. As fitting functions, the Gaussian function and lognormal function use the global information of the signal to extract the characteristics of pulse waves for SAS research (Jiang et al., 2021), but this method requires normalizing the data, resulting in a long processing period. Shen et al. established a convolutional network using PPG signals collected from wearable smart bracelet devices to detect sleep apnea syndrome, but the accuracy of fragment detection is approximately 80% (Shen et al., 2022).

In this study, a one-dimensional convolutional neural network (1D-CNN) was established by using PPG signals for the recognition of SAS, with a classification accuracy of over 90%. The results indicate that the convolutional model based on PPG has satisfactory recognition performance for SAS. This means that SAS can be identified using PPG signals by a one-dimensional convolutional model, which can make the detection process of SAS convenient and comfortable.

## 2. Methods

### 2.1. Subjects and data

In this study, signals were collected from 59 subjects, which included 20 healthy people and 39 SAS patients. The data used for analysis were the PPG signals of the subject’s fingers obtained from the Alice 5 detection system of the polysomnography monitor in the Sleep Center of Shandong Provincial Hospital. It was approved by the committee of our research institute as a retrospective study with the subjects’ informed consent. The PPG signals (sampling frequency is 100 Hz) of each subject are segmented by 1,500 points. Table 1 shows the clinical information of 59 subjects and the summary of the PPG datasets.

**Table 1:** Summary of PPG datasets.

Category	Number	Gender Male subjects /female subjects	Age (years) Mean [range]	Record duration (min) Mean [range]	Data segments
Healthy	20	11/9	29 [21–56]	473 [348–563]	15,343
SAS patient	39	29/10	48 [22–76]	494 [318–557]	20,398
Total	59	40/19	42 [21–76]	486 [318–563]	35,741

As shown in Table 1, this study used 35,741 data segments, all of which were randomly divided into training, validation, and test sets, with a ratio of 6:2:2. To avoid the contingency of the experimental results, five cross-validations were used for training.

### 2.2. One-dimensional convolutional neural network

**Figure 1:**
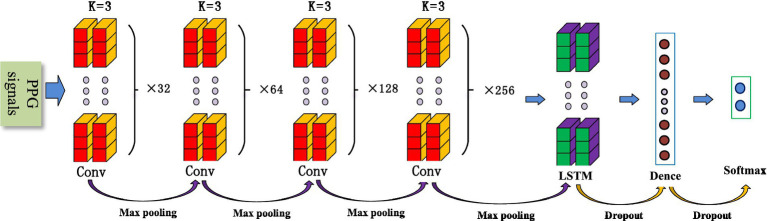
The structure of the 1D-CNN model.

The convolutional neural network (CNN) is a common deep learning model, whose convolutional kernel can extract intrinsic features from different dimensions. It has the characteristics of local perception and weight, allowing the merging of local features from different fields of view. This greatly enhances learning efficiency and accuracy. In addition, its network structure mainly adopts local connections and weight-sharing methods, which reduce the number of weights, facilitate network optimization, and minimize model complexity and the occurrence of overfitting. Considering that pulse wave data is a one-dimensional time series signal, this study proposes a 1D-CNN model for SAS classification and detection that includes eight convolutional layers, four maximum pooling layers, two LSTMs, and two fully connected layers. Figure 1 shows the structure of the 1D-CNN model.

#### 2.2.1. Convolutional layers

Convolutional layers are mainly used for feature extraction and can automatically extract features for learning. Different convolutional kernels can extract different local features, and the amount of feature learning can be increased by setting different convolutional kernels. As the number of layers in a neural network increases, convolutional neural networks typically have stronger feature extraction capabilities and yield better results. However, increasing the number of convolutional kernels significantly raises computational complexity and the difficulty of network training. At the same time, with the increase in network depth, it is easy to cause gradient vanishing and overfitting. To prevent these phenomena and obtain accurate results, this paper designs a progressive convolutional kernel scheme layer by layer. As shown in Figure 1, the model consists of eight convolutional layers, divided into four groups, each with two convolutional layers. A pooling layer and the ELU activation function are added between the convolution layer groups. The k-value of each convolutional kernel is 3, and the number of neurons in the four groups is 32, 64, 128, and 256, respectively.

#### 2.2.2. Pooling layers

Pooling is a process of data processing that reduces the dimensionality of feature maps and the number of parameters in the network. The pooling layer can gradually reduce the feature map output of the network and improve learning efficiency. In this study, four maximum pooling layers were designed. This design achieves rapid dimensionality reduction of information by mapping distributed features to the sample label space while ensuring its comprehensiveness and translation invariance.

#### 2.2.3. LSTM

**Figure 2:**
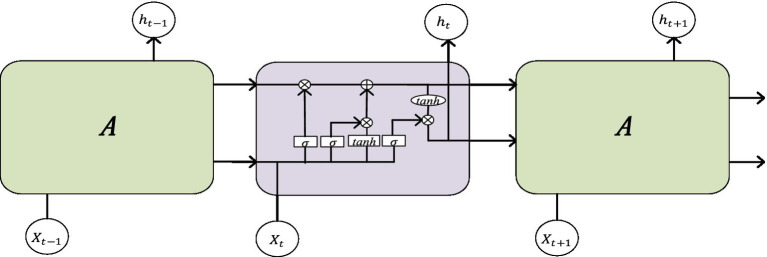
The structural diagram of LSTM.

The Long Short-Term Memory (LSTM) neural network is an improved network based on recurrent neural networks. Due to the fact that traditional RNN structures are prone to associated gradient problems during training, they are not suitable for processing time dependence. The LSTM network can solve the dependency problem of RNN networks through the gate structure, thereby establishing a larger deep network. Its structural diagram is shown in Figure 2. Input gates can facilitate the flow of information and update the state of cells. The output gate can not only achieve information outflow but also be used to determine the value of the next hidden state. The Forgotten Gate can update the previous state and choose whether to discard or retain the information. The sigmoid function categorizes the data between 0 and 1, filters the updated data, and then transfers the output data of the previously hidden layer and the current state data together to the *Tanh* function to determine a new candidate value. Finally, the outputs of these two functions are multiplied.

$$I_{t}=Sigmoid\left(W_{i}.\left[h_{t-1},X_{t}\right]\right)$$

$$F_{t}=Sigmoid\left(W_{f}.\left[h_{t-1},X_{t}\right]\right)$$

$$\tilde{h_{t}}=Tanh\left(W.\left[I_{t}*h_{t-1},X_{t}\right]\right)$$

$$h_{t}=\left(1-F_{t}\right)*h_{t-1}+F_{t}*\tilde{h_{t}}$$

where *I_t_* represents input gates; *F_t_* represents forgetting gates; *h_t_* represents the hidden layer of the output gate; *X_t_* is the external input at the current time; and *h_t-1_* is the output of the network at the previous time.

The pulse wave is a temporal signal, and this article uses two LSTMs to extract temporal features from the data.

#### 2.2.4. Cross-entropy loss function

The loss function is the key factor that guides the optimization direction of neural network parameters. The parameters of the network model are updated according to the backpropagation of the loss function to optimize the model. The cross-entropy loss function uses the logic function to obtain probabilities and adopts an inter-class competition mechanism to effectively learn inter-class information. This scientific question in this paper is a binary problem; therefore, the binary cross-entropy loss function is utilized, which is defined in Formula 1 as:

(1) $$L=-\frac{1}{N}\overset{N}{\underset{i=1}{\sum }}\left[y_{i}.\log\left(p_{i}\right)+\left(1-y_{i}\right).\log\left(1-p_{i}\right)\right]$$

where *N* represents the total number of samples, $y_{i}\;$ represents the label of sample *i*, with positive classes being 1 and negative classes being 0; $p_{i}$ represents the probability that sample *i* is predicted to be positive.

## 3. Results

### 3.1. Evaluation indicators

To validate the performance of the model, four indicators were used to evaluate the classification performance of the model: accuracy (ACC), precision (PRE), sensitivity (SE), and specificity (SP). The calculation formula for each indicator is as follows:

$$ACC=\frac{TP+TN}{TP+TN+FP+FN}$$

$$PRE=\frac{TP}{TP+FP}$$

$$SE=\frac{TP}{TP+FN}$$

$$SP=\frac{TN}{TN+FP}$$

### 3.2. Result comparison

**Figure 3:**
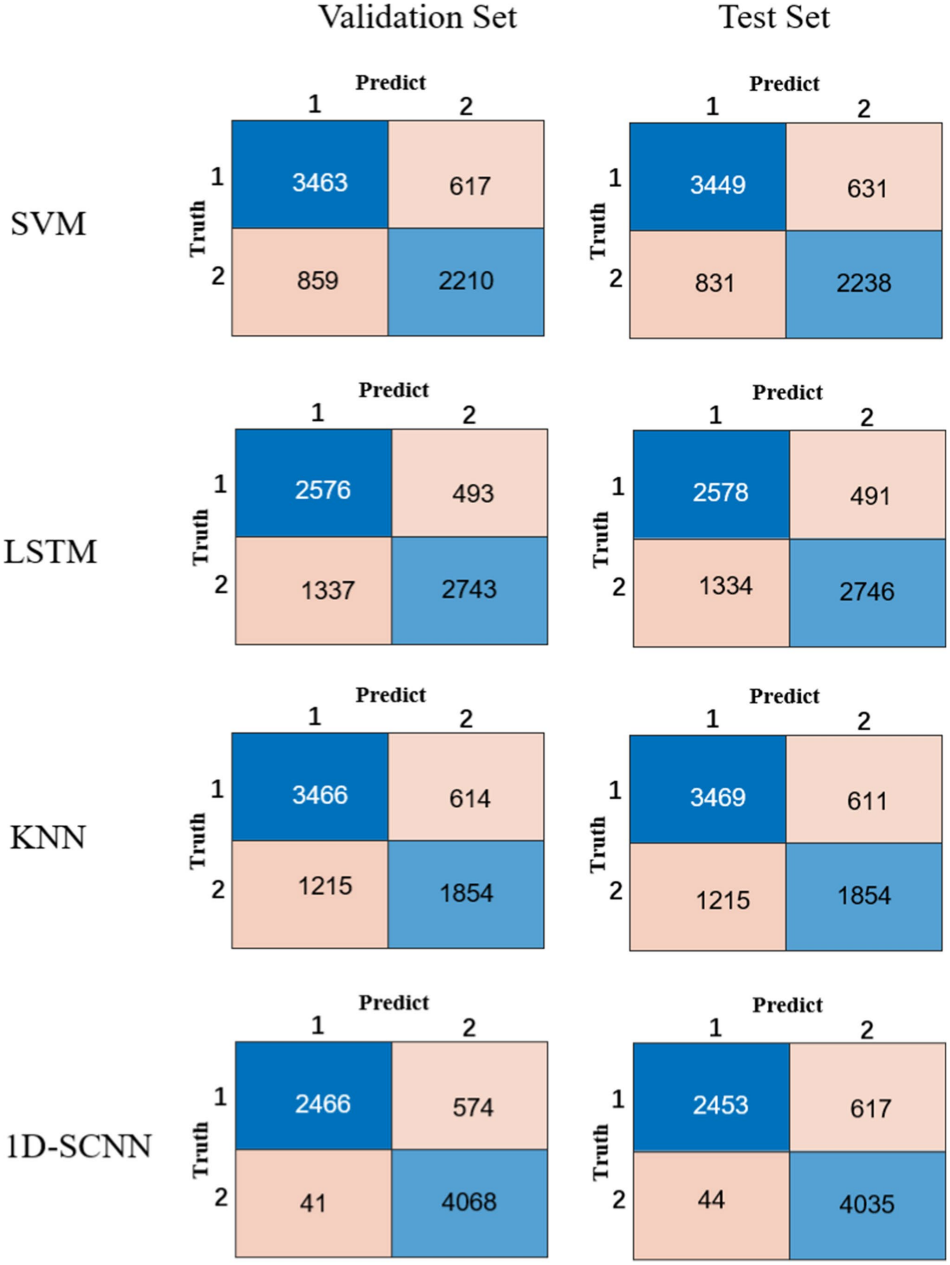
Confusion matrix results of the four models.

In order to verify the performance of the 1D-CNN model, SVM, LSTM, and KNN models were also constructed using the same data. The comparison between the confusion matrix results of the four models is shown in Figure 3. The evaluation index values of each model are shown in Table 2.

**Table 2:** Evaluation index values for each model.

Method	Validation set	Test set						
	ACC (%)	PRE (%)	SE (%)	SP (%)	ACC (%)	PRE (%)	SE (%)	SP (%)
SVM	79.35	84.88	80.12	78.17	79.55	84.53	80.58	78.01
LSTM	74.40	83.94	65.83	84.77	74.47	84.00	65.90	84.83
KNN	74.42	84.95	74.04	75.12	74.46	85.02	74.06	75.21
1D-CNN	**91.40**	**81.12**	**98.36**	**87.63**	**90.75**	**79.90**	**98.24**	**86.74**

Compared to the other three models, it is obvious from Table 2 that the 1D-CNN model established in this paper exhibited good performance. Except for the PRE indicator, the 1D-CNN model achieved the highest values for the other three performance indicators, ACC, SE, and SP Their values are 91.40, 98.36, and 87.63%, respectively, in the validation set; and 90.75, 98.24, and 86.74%, respectively, in the test set.

### 3.3. Anti-interference experiment

To test the influence of noise on the performance of 1D-SCNN, an anti-interference experiment was designed by adding Gaussian white noise to the original signal with a signal-to-noise ratio (SNR) of 5, 10, 15, 20, 25, and 30 dB, respectively. The data segments were also randomly divided into training, validation, and test sets in a ratio of 6:2:2. The anti-interference test results of the model’s test set are shown in Table 3. The experimental results indicated that noise has little effect on the performance indicators of 1D-SCNN, and the model has a certain anti-interference ability.

**Table 3:** Anti-interference test results of the 1D-SCNN model.

SNR	ACC (%)	PRE (%)	SE (%)	SP (%)
5 dB	90.74	79.92	98.16	86.76
10 dB	90.80	80.01	98.20	86.82
15 dB	90.28	79.12	98.08	86.09
20 dB	91.22	80.86	98.21	87.44
25 dB	90.05	78.68	97.91	85.87
30 dB	89.91	78.56	97.63	85.81

### 3.4. Ablation experiment

To verify the role of the LSTM layer in the model, we designed an ablation experiment by removing the LSTM layer from the 1D-CNN model, and then used the original data and followed the same method to train the model without the LSTM layer. The performance indicators ACC, *PRE*, SE, and SP of the test set without the LSTM layer had values of 89.94, 78.57, 97.71, and 85.81%, respectively, which reduced its accuracy by 0.81% compared to the 1D-CNN model. The results showed that the LSTM layer can improve system performance, although the accuracy improvement is not very significant.

## 4. Discussion

In this study, we constructed a 1D-CNN model for SAS detection and compared its performance with SVM, LSTM, and KNN models. The results showed that the accuracy of the 1D-CNN model on the test set was 90.75%, which was 11.2% higher than the results for the SVM model, with a recorded accuracy of 79.55% on the test set. The results indicate that the constrained 1D-CNN model in this study has better performance in the classification of SAS. At the same time, we designed anti-interference and ablation experiments to test the anti-noise performance of the model and the role of LSTM layers, respectively. The experimental results indicated that the model has a certain level of anti-interference ability, and the LSTM layer helps to improve the performance of the model.

Shen et al. (2022) proposed a Multitask Residual Shrinkage Convolutional Neural Network that utilizes PPG signals to detect SAS with a fragment detection accuracy of 81.82%. Lazazzera et al. (2021) also proposed a method to detect and classify sleep apnea and hypopnea using light plethysmography (PPG) and peripheral oxygen saturation [SpO_(2)_] signals. However, there is significant room for improvement in the accuracy of their models. In our previous work (Jiang et al., 2021), Gaussian and lognormal functions were used to build SVM models based on PPG signals to classify SAS. The correct rate of the SVM model with a lognormal function in the awake period reached 95.00%, and the correct rate of the SVM model with a Gaussian function in the rapid eye movement periods reached 93%. However, in this study, only 10 cycles of pulse signals were captured from each subject, and the difference between the number of healthy individuals and the number of patients was too large, while the SVM machine learning method did not separate more subtypes. All these factors make the generalization ability of SVM models weak.

This study has several limitations. First, the sample size is small, involving only 59 subjects for a total of 35,741 data segments, which may have affected the performance of the model. Second, compared to SAS patients, healthy subjects are younger. Previous studies have shown that age affects PPG signals (Millasseau et al., 2002; Liu et al., 2015), and differences in PPG signals caused by different age groups may also affect the classification performance of the model. However, the above factors have a small impact on the performance of the model, which has not changed much overall.

## 5. Conclusion

In this study, a 1D-CNN model based on PPG signals for SAS classification was established. The results showed that this had the best performance, with a test set accuracy of over 90%, compared to other types of models. Our research results indicate that using only PPG signals for SAS classification is feasible, which can provide a foundation for seeking convenient and comfortable SAS detection methods. Furthermore, this can be helpful for portable wearable detection.

## Data availability statement

The raw data supporting the conclusions of this article will be made available by the authors, without undue reservation.

## Ethics statement

Written informed consent was obtained from the individual(s) for the publication of any potentially identifiable images or data included in this article.

## Author contributions

XJ was mainly responsible for data analysis and writing of the manuscript. YR was mainly responsible for data collection and analysis of the manuscript. HW was mainly responsible for algorithms. YL was mainly responsible for organizing data. FL was mainly responsible for the structural design and revision of the manuscript. All authors contributed to the article and approved the submitted version.

## Funding

This work was supported by the National Natural Science Foundation of China (Grant nos. 82072014 and 61901114), the National Key R&D Program of China (Grant no. 2019YFE010670), the Natural Science Foundation of Shandong Province (Grant no. ZR2020MF028), and the Key R&D Program of Shandong Province (Grant no.2020CXGC010110).

## Conflict of interest

The authors declare that the research was conducted in the absence of any commercial or financial relationships that could be construed as a potential conflict of interest.

## Publisher’s note

All claims expressed in this article are solely those of the authors and do not necessarily represent those of their affiliated organizations, or those of the publisher, the editors and the reviewers. Any product that may be evaluated in this article, or claim that may be made by its manufacturer, is not guaranteed or endorsed by the publisher.
